# Lichenysin Production by *Bacillus licheniformis* Food Isolates and Toxicity to Human Cells

**DOI:** 10.3389/fmicb.2022.831033

**Published:** 2022-02-07

**Authors:** Kah Yen Claire Yeak, Manca Perko, Guido Staring, Blanca M. Fernandez-Ciruelos, Jerry M. Wells, Tjakko Abee, Marjon H. J. Wells-Bennik

**Affiliations:** ^1^NIZO, Ede, Netherlands; ^2^Food Microbiology, Wageningen University & Research, Wageningen, Netherlands; ^3^Host-Microbe Interactomics, Wageningen University & Research, Wageningen, Netherlands

**Keywords:** biosurfactant, lipopeptide, hazard, food poisoning, skimmed milk, food safety, Caco-2, spore former

## Abstract

*Bacillus licheniformis* can cause foodborne intoxication due to the production of the surfactant lichenysin. The aim of this study was to measure the production of lichenysin by food isolates of *B. licheniformis* in LB medium and skimmed milk and its cytotoxicity for intestinal cells. Out of 11 *B. licheniformis* isolates tested, most showed robust growth in high salt (1M NaCl), 4% ethanol, at 37 or 55°C, and aerobic and anaerobic conditions. All strains produced lichenysin (in varying amounts), but not all strains were hemolytic. Production of this stable compound by selected strains (high producers B4094 and B4123, and type strain DSM13*^T^*) was subsequently determined using LB medium and milk, at 37 and 55°C. Lichenysin production in LB broth and milk was not detected at cell densities < 5 log_10_ CFU/ml. The highest concentrations were found in the stationary phase of growth. Total production of lichenysin was 4–20 times lower in milk than in LB broth (maximum 36 μg/ml), and ∼10 times lower in the biomass obtained from milk agar than LB agar. Under all conditions tested, strain B4094 consistently yielded the highest amounts. Besides strain variation and medium composition, temperature also had an effect on lichenysin production, with twofold lower amounts of lichenysin produced at 55°C than at 37°C. All three strains produced lichenysin A with varying acyl chain lengths (C11–C18). The relative abundance of the C14 variant was highest in milk and the C15 variant highest in LB. The concentration of lichenysin needed to reduce cell viability by 50% (IC_50_) was 16.6 μg/ml for Caco-2 human intestinal epithelial cells and 16.8 μg/ml for pig ileum organoids. Taken together, the presence of low levels (<5 log_10_ CFU/ml) of *B. licheniformis* in foods is unlikely to pose a foodborne hazard related to lichenysin production. However, depending on the strain present, the composition, and storage condition of the food, a risk of foodborne intoxication may arise if growth to high levels is supported and such product is ingested.

## Introduction

*Bacillus licheniformis* is a facultative anaerobic endospore-producing bacterium that is ubiquitously found in the environment, plant material, and soil. The organism belongs to the *Bacillus subtilis* group and is known for its use in the production of enzymes or antibiotics (e.g., bacitracin), while spore preparations of selected strains are used as crop bioprotectants and feed additives (Wang et al., 2016; Elshaghabee et al., 2017; Radhakrishnan et al., 2017).

*Bacillus licheniformis* is not considered to be a pathogen and has not been shown to be able to invade the outer barriers of the body without previous lesions (e.g., mucosal tissue or skin), but sporadic cases of human infection related to *B. licheniformis* have been reported (Sugar and McCloskey, 1977; Park et al., 2006; Haydushka et al., 2012; Padhi et al., 2012). In cows, the organism has been linked to rare cases of abortion (Johnson et al., 1994; Agerholm et al., 1995) and in a study by Nieminen et al. (2007) high numbers were found in some samples of mastitic milk.

While generally considered safe, *B. licheniformis* has occasionally been reported as a causative agent of foodborne intoxication after the consumption of cooked meat, vegetables, milk powders, and dairy products (Salkinoja-Salonen et al., 1999; Pavic et al., 2005; Logan, 2012; Rønning et al., 2015). Reported cases were characterized by a relatively short incubation time (2–14 h) and high infective dose (>5 log_10_ CFU/g) followed by mild gastrointestinal symptoms (e.g., nausea, stomach cramps, vomiting, abdominal pain, and sometimes diarrhea) lasting for 6–24 h (Kramer and Gilbert, 1989; Salkinoja-Salonen et al., 1999). The agent causing foodborne illness was identified as the surfactant lichenysin (Salkinoja-Salonen et al., 1999; Mikkola et al., 2003) with one fatal case linked to the consumption of infant formula containing lichenysin (Salkinoja-Salonen et al., 1999).

Spores of *B. licheniformis* may be present in food ingredients and survive commonly applied heating processes such as pasteurization. The heat resistance of spores can vary significantly between isolates: some carry genetic elements that give rise to the production of high-level heat-resistant spores (Berendsen et al., 2016a). Surviving spores can germinate, return to their vegetative state, and then grow in the finished food, depending on the composition of the product and its storage condition. The organism tolerates a relatively low water activity (A_*w*_) of ∼0.9 and a broad temperature range (∼10°C – 58°C) for growth (Warth, 1978; Baranyi and Tamplin, 2004). This includes food processing conditions at relatively high temperatures, for instance in evaporators that are operated at temperatures around 55°C. *B. licheniformis* can be the predominant spore former in milk powders made from pasteurized milk that is evaporated and spray-dried (Miller et al., 2015; Eijlander et al., 2019), even though the raw milk used to make powder contains a broad range of spore-forming species (Coorevits et al., 2008; Miller et al., 2015). High temperatures in evaporators (operated under vacuum) in combination with a low Aw in the concentrated product stream put selective pressures that favor outgrowth and subsequent spore formation by *B. licheniformis* (Burgess et al., 2010; Eijlander et al., 2019; Delaunay et al., 2021). Besides dairy products, the organism is also frequently found in spices, dry herbs (e.g., pepper and turmeric) (Mathot et al., 2021), and flours (Rosenkvist and Hansen, 1995; Iurlina et al., 2006). As a common contaminant in food ingredients, the processing conditions will determine whether viable spores are present in the finished product (Postollec et al., 2012). If the product and storage conditions subsequently support the growth of *B. licheniformis*, a possibility of lichenysin production in the food exists (Warth, 1978; Postollec et al., 2012).

Lichenysin is a secondary metabolite. It is synthesized by the proteins encoded by the *lchAA-AB-AC-TE* gene cluster, also annotated as *licA-TE* (Konz et al., 1999). The biosynthesis of the peptide fraction of lichenysin is catalyzed by non-ribosomal peptide synthetases (NRPS). The presence of *lch* genes in *B. licheniformis* appears to be very common: the *lchAA* gene was detected by PCR in all 53 isolates studied by Madslien et al. (2013). The whole-genome sequences of these isolates are not available but lichenysin production was confirmed for all strains in their study. The presence of the *lch* cluster in a particular strain does not necessarily mean that the compound is produced. This depends on the level of transcription, translation, and enzyme (lichenysin synthetase) activity level (Harwood et al., 2018). Environmental conditions, such as the type of carbon, nitrogen, or phosphate sources present in the environment strongly influence production (Coronel-León et al., 2015b).

**FIGURE 1:**
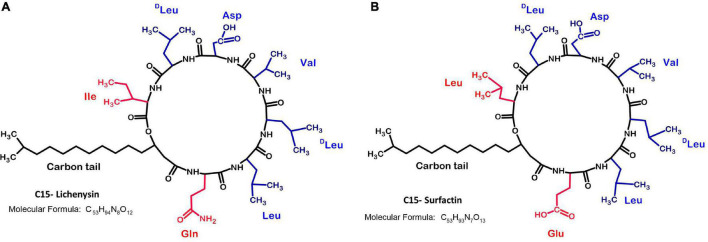
Chemical structures of C15-lichenysin and C15-surfactin. **(A)** C15 Lichenysin; **(B)** C15- Surfactin; The lipopeptide lichenysin A (cyclo-[Gln^1^-Leu^2^-Leu^3^-Val^4^-Asp^5^-Leu^6^-Ile^7^ − −β-OH fatty acid]) produced by *B. licheniformis* (Yakimov et al., 1995) is structurally similar to surfactin (cyclo-[Glu^1^-Leu^2^-Leu^3^-Val^4^-Asp^5^-Leu^6^-Leu^7^ − − β-OH fatty acid]) produced by *B. subtilis* (Zhao et al., 2017), which was first discovered by Arima et al. (1968). The 1st and 7th position of the peptide ring differs between surfactin and lichenysin A.

Lichenysin is an amphiphilic lipopeptide. Its structure is highly similar to that of surfactin, a well-known surfactant produced by *B. subtilis* (Arima et al., 1968; Figure 1). Both compounds can be produced aerobically or anaerobically (Javaheri et al., 1985; Willenbacher et al., 2015; Coronel-León et al., 2016a; Hoffmann et al., 2020). They consist of a hydrophilic peptide ring of seven amino acids, connected to a hydrophobic β-hydroxy fatty acid chain (Joshi et al., 2008; Coronel-León et al., 2016a,b). The β-hydroxy fatty acids can vary in length between 12 and 17 carbons and can be normal or branched in *iso* or *anteiso* forms (Yakimov et al., 1995). Lichenysin A contains glutamine (Gln) and isoleucine (Ile) on the 1st and 7th position of the cyclic peptide, respectively, whereas surfactin has glutamate (Glu) and leucine (Leu) on these positions (Figure 1). Other lichenysin isoforms are more similar to surfactin, e.g., lichenysin B only differs in the fatty acid chain, and lichenysin C has one amino acid difference in the peptide ring (Leu > Ile) (Nerurkar, 2010). Lichenysin D and G isoforms are almost identical to lichenysin A, with just one amino acid difference at the 7th position; lichenysin D has either Leu or Valine (Val), whereas lichenysin G has Val (Nerurkar, 2010).

The various isoforms of these surfactants share similar features such as good solubilizing, foaming, emulsifying, and detergent activity (Rajendran and Marahiel, 1999; Zhao et al., 2017; Santos et al., 2018; Thakur et al., 2020), but they can have different physicochemical and bioactive properties, depending on the amino acids in the peptide ring and the length and type of the fatty acid chain (Santos et al., 2018). Lichenysin A, for instance, has higher surfactant power than surfactin, likely due to the two different amino acids in the peptide ring, and it has 2 to 10-fold lower critical micelle concentrations (CMC) than surfactin (Grangemard et al., 2001; Nerurkar, 2010). The multifaceted properties of these amphiphilic lipopeptides account for their broad range of applications, such as biocontrol in the agricultural industry (Henry et al., 2011; Sachdev and Cameotra, 2013), emulsifiers in the oil industry (Yao et al., 2021), foaming agents in cosmetics (Varvaresou and Iakovou, 2015), and detergents in household cleaning products (Shaligram and Singhal, 2010).

Once produced, lichenysin is highly stable under extreme conditions (Coronel-León et al., 2015a,b). Its surfactant properties are unaffected over a wide pH range (pH 6–11), in the presence of high salt concentration (∼ up to 20% NaCl), and upon exposure to temperatures as high as 121°C (Coronel-León et al., 2015b; Purwasena et al., 2019). Next to its action as a surfactant, lichenysin is also a good ion chelator, exerts antibiotic activity, interacts with phospholipids, and induces the formation of ion channels in artificial membranes (Grangemard et al., 2001; Coronel-León et al., 2017).

Production of lichenysin in foods may pose a food safety risk. It has hemolytic activity and toxic effects on boar spermatozoa cells at concentrations above 10 μg/ml, and on Vero cells at concentrations above 33 μg/ml (Madslien et al., 2013), whereas toxicity data for human-derived cell lines have not been reported. This activity is tightly linked to the capacity of lichenysin to interact with cell membranes and disrupt membrane barrier functions (Coronel-León et al., 2017). Although lichenysin A production by *B. licheniformis* in food has been connected with cases of food poisoning (Salkinoja-Salonen et al., 1999; Mikkola et al., 2003; Nieminen et al., 2007; Logan, 2012; Madslien et al., 2013; Rønning et al., 2015), data on amounts and types of lichenysin synthesized during growth in food matrices, levels produced at different stages of growth, and dependency on culturing conditions are not available.

In this study, we determined the types and amounts of lichenysin produced by selected *B. licheniformis* food isolates in nutrient-rich Luria Bertani (LB) medium and skimmed milk at different temperatures. Additionally, we assessed the cytotoxicity of lichenysin with surfactin as a reference.

## Materials and Methods

### Bacterial Strains and Culture Conditions

The following foodborne isolates of *B. licheniformis* from the NIZO culture collection were used: B4089, B4090, B4091, B4092, B4094, B4121, B4123, B4124, B4125, and B4164. Details related to their isolation sources and whole-genome accession numbers were reported (Berendsen et al., 2016a). Genomes were checked for the presence of the lichenysin gene cluster (*licA, licB, licC*, and *licTE*) using the Benchling biology software, available at https://benchling.com. *B. licheniformis* DSM13*^T^* type strain was obtained from the German Collection of Microorganisms and Cell Cultures, Braunschweig, Germany (DSMZ). Bacteria were routinely cultured using LB medium (Bertani, 2004) (Tritium Microbiologie BV, Eindhoven, Netherlands) and incubated aerobically at 37°C, using shaking at 220 rpm, unless stated otherwise.

### Screening and Selection of *Bacillus licheniformis* Food Isolates

#### β – Hemolytic Activity on Columbia Blood Agar

To screen strains for hemolytic activity, the Columbia blood agar method (Mulligan et al., 1984) was used as described by Madslien et al. (2013) with minor adjustments. Briefly, the optical densities (OD_600_) of the overnight (ON) cultures in LB were measured and adjusted to OD_600_ 2.0. Ten μl of each culture was spotted on Columbia agar base supplemented with 5% sheep blood (Tritium Microbiologie BV) and incubated at 37°C. The hemolytic activity of each strain was inspected after 24 and 48 hours (h) of incubation. Strains that caused lysis of the red blood cells exhibited a transparent clearing zone around the colonies, indicating the presence of the biosurfactant lichenysin.

#### Oil Displacement Test

To assess the ability of *B. licheniformis* strains to produce biosurfactants, the oil displacement method as described by Walter et al. (2013) was used with modifications. Briefly, the oil used in this study was a mixture of linseed: wheat germ: sesame oils in the ratio of 3: 0.5: 0.05 (Mitterzumleben, Oberthal, Germany). One ml of the oil mixture was evenly placed on top of 3.5 ml of distilled water in a 6-wells plate (Thermo Fischer Scientific, Bleiswijk, Netherlands). An aliquot of 10 μl of an ON culture of *B. licheniformis* was dropped on the oil layer, and the diameter of displaced oil was measured.

#### Aerobic and Anaerobic Growth in 96-Wells Plate

An ON culture of *B. licheniformis* in LB was adjusted to OD_600_ 0.05 in fresh LB medium and grown to OD_600_ ∼0.5 at 37°C. Then 2 μl was added to either 198 μl of LB medium, or LB medium containing 1 M NaCl, or LB containing 4% ethanol (v/v) in 96 well plates (Greiner Bio-One B.V, Alphen aan den Rijn, Netherlands). Two different plates were incubated at 37 or 55°C in the Epoch2 microplate reader (Biotek, Hellevoetsluis, Netherlands) in dual orbital shaking mode, and growth was followed for ∼20 h by measuring the OD_600_.

For anaerobic growth, all lab materials and cultivation media were transferred into an anaerobic chamber (model #830 -ABE/OTA) (Plas-Labs, Lansing, MI, United States), 24 h before the experiment. The gas mixture used was 5% H_2_, 5% CO_2_, 90 % N_2_. All strains were first cultured to an OD_600_ ∼ 0.5 aerobically, then 2 μl of cultures were added to 198 μl of anaerobic LB medium containing 0.2% of potassium nitrate (KNO_3_). Growth was followed by monitoring the OD_600_ for ∼20 h using the Epoch2 microplate reader (BioTek) inside the anaerobic chamber. Two μl of the blue fluorogenic dye Resazurin (1 g/l in distilled water) (Merck, Zwijndrecht, Netherlands) was added to an additional well that contained (198 μl) cell culture and was used as a redox indicator. The color change from blue to pink and then to colorless indicated the absence of oxygen.

### Growth Conditions and Sample Collection for Lichenysin Extraction

#### Growth Curves in LB Broth and Skimmed Milk

Two selected *B. licheniformis* food isolates B4123, B4094, and the type strain DSM13*^T^* were cultured either in LB or skimmed milk (Tritium Microbiologie BV) ON at 37°C, 220 rpm. The OD_600_ of ON cultures was measured and adjusted to OD_600_ 0.05, diluted sixfold, resulting in around 10^2^ colony-forming units (CFU) ml^–1^ in 100 ml of fresh prewarmed medium. Samples were collected at time point 0 (T0) and subsequently, cultures were incubated at 37°C, with continuous shaking (220 rpm) for 72 h. During the growth of these cultures, samples were collected to determine the CFUs in time and to determine the concentrations of lichenysin in the culture.

In LB, samples were collected every 2 h for the first 24 h and for the first 28 h in skimmed milk to determine the CFUs in time. Lichenysin produced in the culture was determined at every 4 h in LB and skimmed milk, with additional selected time points in between the 4 h interval after cells reached the early stationary phase. Aliquots of 5 ml cell cultures were collected and frozen immediately at –20°C until used for lichenysin extraction.

At each mentioned time point, 1 ml of the culture was 10-fold serially diluted using sterile phosphate-buffered saline (PBS), and appropriate dilutions were plated out either on LB agar or Plate count skimmed milk agar (PCMA) (Tritium Microbiologie BV) followed by incubation at 37°C ON and counting of the colonies. The CFU/ml at each time point for each strain was determined using the formula (Number of colonies × dilution factor) / volume spread on the plate. In addition, lichenysin concentrations in the cultures at each time point were analyzed and quantified (see sections “Lichenysin Extraction”).

To measure the OD_600_ of ON cultures in skimmed milk, the milk was cleared by mixing 1 ml of ON culture with 9 ml of citrate milk clearing solution (2%) (Tritium Microbiologie BV). For growth in skimmed milk, two additional samples were collected at time points 26 and 28 h as all three strains grew slower in skimmed milk than in LB and cultures reached the stationary phase later than in LB. Incubation was performed at 37°C.

#### Estimation of Lag Time, Lambda (λ) and Specific Growth Rate, Mu (μ)

The average log_10_ CFU/ml for the strain B4094, B4123, and DSM13, respectively, in LB and skimmed milk from two independent experiments were calculated. Their respective specific growth rate (mu/μ) and lag time (lambda/λ) for each strain/medium were estimated using the Baranyi primary growth model (Baranyi and Roberts, 1994; Buchanan et al., 1997), performed using the Github version of the Biogrowth R package. The Biogrowth R package is available at the Comprehensive R Archive Network (CRAN)^1^ and the Github page at https://github.com/albgarre/biogrowth_web. The Baranyi model was selected in this study because it is known to have the lowest variability for estimating growth rates in comparison to Gompertz, Richards, and the logistic model (Pla et al., 2015).

#### Surface Growth on LB and Skimmed Milk Agar and Sample Collection for Endpoint Lichenysin Quantification

To compare lichenysin produced in diverse environments (liquid broth versus solid surface), B4094, B4123, and DSM13*^T^* were cultured on LB and skimmed milk agar. ON cultures in LB or skimmed milk of the three selected strains were used to inoculate fresh LB broth or skimmed milk (15 ml) to an OD_600_ of 0.05, and cultures were grown to an OD_600_ of 0.5 by incubation at 37°C with agitation (200 rpm). Aliquots of 500 μl of the cultures that were grown in LB or skimmed milk were then spread evenly on the LB and skimmed milk agar plates containing 2% agar, respectively, and allowed to dry under the laminar flow hood. All plates were sealed with parafilm and incubated at 37°C for 10 days, after which biomass was collected from the agar surface using a sterile scraper. The collected biomass (150 mg) was weighed inside an empty soda-lime glass tube (DWK Life Sciences, Staffordshire, United Kingdom), and stored at –20°C until used for lichenysin extraction.

### Lichenysin Extraction

#### Extraction From Liquid Culture Samples During Growth in LB and Skimmed Milk

Lichenysin was extracted as described by Madslien et al. (2013) and Rønning et al. (2015) with slight modifications. Briefly, a 5 ml aliquot of the collected liquid cultures (see section “Growth Curves in LB Broth and Skimmed Milk”) was mixed with 5 mL of pure methanol (Merck) in a soda-lime glass tube (DWK Life Sciences), and vortexed for 5 min until a homogenous mixture was obtained. The mixture was heated for 30 min at 80°C in a water bath. At 10 min intervals, each tube was vortexed for 20 s. Subsequently, the mixture of methanol and cells was cooled down for 20 min on the bench, and centrifuged for 10 min at 4000 *g* at room temperature. The liquid phase was transferred to a new glass tube. The cell pellet was subsequently resuspended in 2 ml of methanol, and the extraction process was repeated. Per sample, the liquid phases collected from the first and second extraction procedures were mixed and evaporated under N_2_ flow at 40°C in a heating block. The dry residue was resuspended in 500 μl of methanol and lichenysin was quantified in these samples.

#### Extraction of Lichenysin From Biomass Collected From Surface Growth on LB Agar and Skimmed Milk Agar

Extraction of lichenysin from biomass was performed as described above for the liquid culture extractions. Three ml of pure methanol was added to 150 mg of cell biomass weighted in a soda-lime glass tube (DWK Life Sciences) and vortexed for 5 min until a homogenous mixture was obtained. The second extraction was performed using 1 ml of methanol. After evaporation, the dry lichenysin extract was resuspended in 150 μl of methanol before quantification.

#### Quantification of Lichenysin by Reversed-Phase High-Performance Liquid Chromatography – Electro Spray Ionization Mass Spectrometry

Qualitative and quantitative analyses of lichenysin A variants as extracted from either liquid cultures or biomass were performed using Reversed-Phase High-Performance Liquid Chromatography (RP-HPLC) with high-resolution accurate mass spectroscopic (MS) detection. All chemicals and reagents used were HPLC grade (Merck). Surfactin (≥98.0% purity with >99% consisting of the C15 variant, HPLC grade) (Merck) and lichenysin ≥ 90.0% purity (Lipofabrik, Villeneuve-d’Ascq, France) were used as standards in the quantification.

The instrumentation used for RP-HPLC was the Agilent 1260 SL system (Agilent Technologies, Middelburg, Netherlands) consisting of a degasser, binary pump, thermostatted autosampler, and column compartment. Aliquots of 5 μl of lichenysin samples were injected onto a Kinetex C8 Minibore column (2.6 μm, 100 Å, 100 × 2.1 mm, p/n: 00D-4497-AN) (Phenomenex, Utrecht, Netherlands), and separation was performed at 50°C. The temperature of the autosampler was 10°C. Lichenysin A was eluted at a flow rate of 0.2 ml/min with a linear gradient of 0.10% formic acid in 40% water + 55 % acetonitrile + 5% tetrahydrofuran (mobile phase A) to 0.1% formic acid in 75% acetonitrile + 25% tetrahydrofuran (mobile phase B) in 20 min.

The mass spectrophotometer (Agilent Technologies) comprised a high-resolution accurate mass quadrupole time of flight G6530B, equipped with a dual Electrospray Ionization (ESI) source, operated in 2 GHz, in extended dynamic range mode. The ionization was performed in positive mode with reference mass correction. The fragmentor voltage was 150 V, gas temperature 350°C, drying gas flow 10 l min^–1^, nebulizer pressure 25 psi, capillary voltage 3500 V, and collision energies 25 psi.

A calibration curve of lichenysin was made for external calibration based on lichenysin standards in acetonitrile (14 different concentrations in the range of 10 – 40,000 μg/l) from a stock concentration of 5 mg/ml (Lipofabrik).

The LCMS was operated in a full-scan mode to scan the parent ions (M + xH)*^x+^* from the range of 100 to 2500 m/z. The most abundant ion representing lichenysin A in the standard was [M + H]+ 1021.6908 (m/z), similar to the description by Madslien et al. (2013) and Rønning et al. (2015). Lichenysin A and its variants in the samples were identified according to the masses listed in Supplementary Table S1. Quantitative data were acquired in the positive detection mode, and data analysis was done with MassHunter Qualitative and Quantitative Analysis software version B.07.00 (Agilent Technologies). The Limit of detection LOD (= Limit of quantification) was 0.005 μg/ml.

### Cytotoxicity Assay

#### Preparation of Concentrated Lichenysin Extract

To perform cytotoxicity assay, two batches of concentrated lichenysin were prepared from biomass grown on LB agar plates as described in section “Extraction of Lichenysin From Biomass Collected From Surface Growth on LB Agar and Skimmed Milk Agar.” Instead of using 150 mg biomass, lichenysin was extracted from 6.5 g of biomass from strain B4123, and dissolved in a final volume of 300 μl methanol. A small aliquot of 1 μl was taken to determine the concentration of the concentrated lichenysin extract *via* Reversed-Phase High-Performance Liquid Chromatography – Electro Spray Ionization Mass Spectrometry (RP-HPLC-ESI-MS). The remaining concentrated extract was entirely evaporated under a flow of nitrogen at 40°C in a heating block. The dry residue was resuspended in either 99% Dimethyl Sulfoxide (DMSO) or Dulbecco’s Modified Eagle Medium (DMEM; Gibco, Thermo Fischer Scientific, Bleiswijk, Netherlands) without phenol red serum or antibiotics. Surfactin (purchased from Merck) was also used in cytotoxicity assays by dissolving 10 mg in 1 ml of distilled water (10 mM) (with an adjusted pH of ∼11.7 to obtain full solubility). Both lichenysin and surfactin were diluted to the desired concentration in DMEM medium without phenol red, serum and antibiotics before adding to the cells.

#### Propagation and Culturing of Human Cell

Human Embryonic Kidney (HEK293) cells were obtained from Invivogen (San Diego, CA, United States), and Caco-2 human intestinal epithelial cells (ATCC HTB-37) were purchased from the American Type Culture Collection (Manassas, VA, United States). Both cell lines were maintained in DMEM medium containing 10 % Fetal Bovine Serum (FBS) (Thermo Fischer Scientific) and 1 % penicillin/streptomycin (Merck) to attain a confluent growth. The grown HEK293 and Caco-2 cells were seeded and suspended at a final concentration of 2 × 10^5^ cells/ml (in 200 μl) in 96 wells-plates (Greiner). The 96 wells-plates were then incubated for 24 h at 37°C, under a gas atmosphere of 5% CO_2_ for cell adherence, and washed with PBS to remove unbound cells. The attached cells were exposed to different concentrations (0–200 μM) of surfactin [diluted with distilled water (pH 11.7) from a 10 mM stock] or lichenysin (dissolved in DMEM, prepared as described in “Extraction of Lichenysin From Biomass Collected From Surface Growth on LB Agar and Skimmed Milk Agar”) for a period of 72 h.

##### Generation of 3D Pig Ileum Organoids

Pig ileum organoids were generated by the method described by Sato et al. (2011) using modifications described by van der Hee et al. (2018, 2020). Briefly, 3D organoids were dissociated into single cells by TrypLE digestion and seeded in a 96-well plate, and incubated until reaching full confluency. Subsequently, cells were exposed to a range of 0 – 200 μM of surfactin diluted with distilled water (pH ∼11.7) from a 10 mM stock or lichenysin (dissolved in DMSO, prepared as described in “Extraction of Lichenysin From Biomass Collected From Surface Growth on LB Agar and Skimmed Milk Agar”) for a period of 72 h.

##### AlamarBlue Assay

The alamarBlue (Thermo Fischer Scientific) fluorometric assay was used to examine the cytotoxicity of lichenysin and surfactin toward two human cell lineages (HEK293, Caco-2) and ileum organoids from pigs. Culturing medium DMEM with Alamar blue (i.e., a no-cells control) and cells exposed to 10% DMSO were used to correct for background fluorescence and cell death, respectively. To assess cell viability or death upon exposure to surfactin or lichenysin, cells without treatment were used as a negative control. In the cytotoxicity assessment of surfactin and lichenysin toward pig ileum cells, cells treated with 1% DMSO instead of cells without treatment were used as a negative control because lichenysin was dissolved in DMSO instead of the DMEM medium.

The alamarBlue assay was performed as described in Burczynski et al. (2000), Hamid et al. (2004), and Rampersad (2012). Briefly, the Alamar Blue solution was added to a final concentration of 10 % (v/v) in each well. The plates were incubated for 4 h and exposed to an excitation wavelength of 530 nm. The fluorescence at the emission wavelength of 590 nm was measured with a SpectraMax M5 (Molecular Devices, San Jose, CA, United States) and expressed as relative fluorescence units. The average fluorescence of the no-cells control was used to normalize all recorded fluorescence emission values.

The cell viability (%) was calculated by using the formula:


(Fs-FavgnocellscontrolFavguntreatedcellsORFavgcellstreatedwith 1%DMSO)×100

F_*s*_, the recorded fluorescence for cells in each well after the exposure to surfactin or lichenysin;

F_*avg*_ no cells control, the average fluorescence recorded for the no-cells control;

F_*avg*_ untreated cells, the average fluorescence recorded for the untreated cells;

F_*avg*_ 1% DMSO, the average fluorescence recorded for the cells treated with 1% DMSO.

## Results

### Initial Screening Experiments for the Growth of 11 *Bacillus licheniformis* Food Isolates and Lichenysin Production in Different Conditions

The growth of 10 food isolates of *B. licheniformis* (B4089, B4090, B4091, B4092, B4094, B4121, B4123, B4124, B4125, B4164) and type strain DSM13*^T^* under various conditions are presented in Table 1. The underlying growth data are presented in Supplementary Figures S1–S4. In addition, all strains were screened for hemolytic activity (Supplementary Figure S3), and the ability to produce lichenysin *via* RP-HPLC-MS (Table 1 and Supplementary Table S2).

**TABLE 1:** Properties of *Bacillus licheniformis* food isolates and the type strain DSM13*^T^*.

Medium	Blood agar	Growth in LB broth^c^						Growth on LB agar^d^	LB agar
Strain^a^	Hemolysis^b^	37°C	55°C	1M NaCl, 37°C	1M NaCl, 55°C	4% EtOH, 37°C	0.20%KNO_3_ 37°C	60°C	Lichenysin production^e^
B4089	Weak	+	+++	+ +	+	–	–	NT	+
B4090*	None	+ + + +	+++	+ + + +	+ +	+ + +	+	+ + +	+
B4091	None	+ + + +	+ + +	+ +	+ +	+ + + +	+	+ + +	+
B4092*	Weak	+ + + +	+ + +	+ +	+ +	+ + +	-	+ +	+
B4094*	**Weak**	+ + + +	**+ + +**	**+ + + +**	**+ +**	**+ + + +**	**++**	**+ + + +**	+
B4121	Strong	+ +	+ +	+ + +	+	+ + + +	+	+	+
**B4123**	**Super strong**	**+ + + +**	**+ + +**	**+ + +**	**+**	**+ +**	**+**	**+**	+
B4124	Weak	+ + + +	+ + +	+ +	+ +	+ +	+	+ +	+
B4125	Medium	+ + +	+	+ + +	+	+ +	+	+	+
B4164	None	+ + +	+	+ +	+ +	+ +	-	+	+
**DSM13*^T^***	**None**	**+ +**	**+ +**	**+ +**	**+ +**	**+ +**	**+**	**+ + +**	+

^a^Food isolates sources as indicated in Berendsen et al. (2016b). * Indicates isolates that are heat resistant with Tn1546 transposons encompassing the spoVA^2mob^ operons. DSM13^T^ type strain was purchased from the German Collection of Microorganisms and Cell Cultures, Braunschweig, Germany (DSMZ).

*^b^Hemolysis test carried out on Columbia blood agar plates; None, no clearing zone was observed; Weak, very small clearing zone; medium, medium size clearing zone; strong, big clearing zone; Super strong, very big clearing zone (Supplementary Figure S3).*

*^c^Growth data measured in OD_600 nm_; Minus (–) = no growth; + sign indicates growth robustness based on OD_600_ measurement (Supplementary Figures S1, S4).*

*^d^Growth data at 60°C was checked on LB agar via spot platting of 3 μl of OD_600_ 1.0 day culture. NT = not tested; ++++, growth observed in 10^–9^ dilution; +++, growth observed > 10^–7^ but <10^–9^ dilution; ++, growth observed in dilution > 10^0^ but <10^–7^; +, growth only observed at 10^0^ dilution (Supplementary Figure S2).*

*^e^Lichenysin production screening test via RP-HPLC ESI-MS; no quantification data; positive, lichenysin peak was detected (Supplementary Table S2).*

*Bold font, three selected strains for further experiments in this study.*

All 11 isolates showed comparable growth at 37°C in LB broth, except strain B4089 that grew more slowly. All isolates grew at 55°C in LB broth, a temperature close to their reported maximum growth limit of 58°C (Warth, 1978; Berendsen et al., 2016b; Table 1 and Supplementary Figure S1). Strains B4094, B4090, and B4091 showed growth at an even higher temperature, at 60°C, with B4094 growing the best with the highest cell count (see Table 1 and Supplementary Figure S2). The presence of high salt concentrations of 1M NaCl in LB broth (resulting in an A_*w*_ of ∼0.927 – 0.935; Gekas et al., 1998) did not have a substantial impact on the final OD_600_ that was reached by the cultures of all 11 tested isolates at 37°C, while at 55°C the additional salt stress resulted in lower cell densities (Supplementary Figure S1). Growth of all isolates in the presence of 4% (v/v) ethanol in LB was observed except for B4089. Under anaerobic conditions with 0.2% KNO_3_ at 37°C, three isolates (B4089, B4092, B4164) of 11 isolates failed to grow, and B4094 showed the most growth.

The hemolytic activity of all isolates on the Columbia blood agar varied substantially (Table 1 and Supplementary Figure S3). Out of the 11 tested strains, three food isolates (B4090, B4091, B4164) and the DSM13*^T^* strain did not show hemolytic activity, while obvious clearing zones were formed on blood agar plates by the other eight strains. Strains B4089, B4092, B4094, B4124 showed weak hemolytic activity, strains B4125 and B4121 showed medium and strong hemolytic activity, respectively, and strain B4123 showed the strongest hemolytic activity (Table 1 and Supplementary Figure S3).

All strains showed the ability to produce lichenysin as detected by RP-HPLC-MS in biomass obtained from LB agar at 37°C (Supplementary Table S2), with reference strain DSM13*^T^* producing the lowest amounts. The complete lichenysin gene cluster (*lchAA-TE*, also annotated as *licA-licTE*) was found in 10 of 11 strains, based on analysis of the published genomes (Berendsen et al., 2016b). The available genome sequences of strain B4089 showed the presence of *lchAC* (*licC*) and *lchTE* (*licTE*) but not *lchAA (licA)* and *lchAB (licB)*. The presence of both genes was detected in this strain based on PCR analysis (using *lchAA* and *lchAB* specific primers and B4089 chromosomal DNA as a template; data not shown) (Supplementary Table S3). Based on lichenysin production and the positive PCR results, the available genome sequence of B4089 may be incomplete.

#### Growth and Lichenysin Production in Strains B4094, B4123, and DSM13*^T^*

The growth and lichenysin production under different conditions was further assessed for three selected strains, namely: B4094 which showed robust growth in the presence of ethanol and NaCl, growth at 60°C, and produced heat-resistant spores (Berendsen et al., 2016b); B4123 which showed strong hemolytic activity and intermediate growth in all tested conditions; and DSM13*^T^* which was used as a reference strain.

**FIGURE 2:**
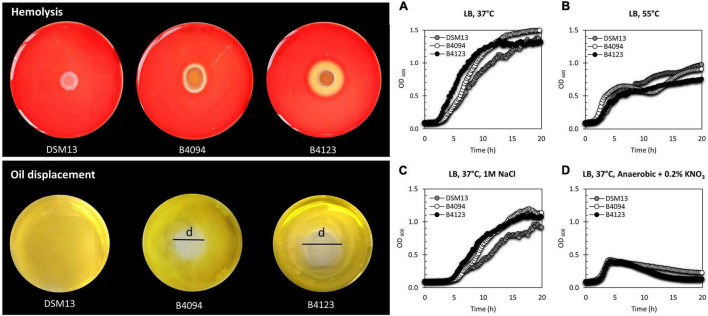
Hemolysis, oil displacement, and representative growth data for the three selected strains, B4094, B4123, and DSM13*^T^*. (Top left) DSM13*^T^*, B4094, and B4123 growth on Columbia blood agar showing hemolytic activity. The clearing zone on the blood agar indicates results from lysis of erythrocytes, indicative of the presence of the biosurfactant lichenysin. (Bottom left) The oil displacement activity of DSM13*^T^*, B4094, and B4123. The displacement zone (d) shows the oil-repelling movement away from water, indicative of the presence of a surfactant. (Right) Aerobic and anaerobic growth data for DSM13*^T^* (gray), B4094 (white), and B4123 (black) in LB medium. **(A)** Growth in LB at 37°C; **(B)** Growth in LB at 55°C; **(C)** Growth in LB + 1M NaCl at 37°C; **(D)** Anaerobic growth in LB + 0.2% KNO_3_ at 37°C.

A direct comparison of the hemolytic activity of the strains and the growth under different conditions is presented in Figure 2, with B4123 showing strong hemolytic activity (large clearing zone), B4094 forming a small clearing zone, while DSM13*^T^* was not hemolytic. DSM13*^T^* did not displace oil from water, but both B4094 and B4123 showed clear displacement activity (Figure 2). In view of the potential of DSM13*^T^* to produce lichenysin (see previous section), these results indicate that B4094 and B4123 produce higher amounts of lichenysin than DSM13*^T^*, resulting in the observed hemolytic activity of erythrocytes and oil displacement.

When grown in LB at 37°C, food isolates B4094 and B4123 grew better than DSM13*^T^* (Figure 2A), whereas, at 55°C, strains B4094 and DSM13*^T^* reached a higher cell density than B4123 (Figure 2B). Under high salt concentration (1M NaCl), both food isolates showed a growth advantage over DSM13*^T^* but did not differ evidently from one another (Figure 2C) and all three strains showed growth under anaerobic conditions in the presence of nitrate (Figure 2D).

### Growth and Lichenysin Production by Selected Food Isolates B4094, B4123, and DSM13*^T^* in LB Broth and Skimmed Milk

#### Growth in LB Broth and Skimmed Milk

**FIGURE 3:**
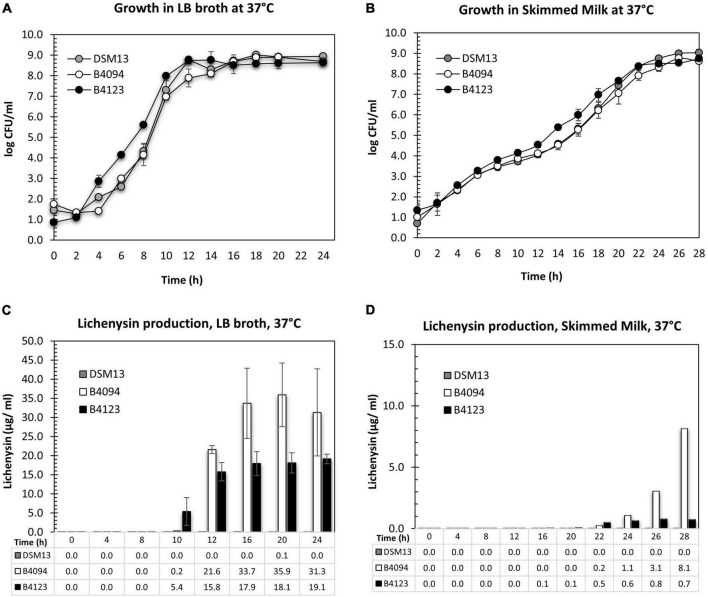
Growth and lichenysin production for DSM13*^T^*, B4094, and B4123 in LB and skimmed milk at 37°C. **(A)** Growth of three selected strains, DSM13*^T^*, B4094, and B4123 in LB medium for 24 h. **(B)** Growth of three selected strains, DSM13*^T^*, B4094, and B4123 in skimmed milk for 28 h. **(C)** Total lichenysin production (μg/ml) in LB broth for DSM13*^T^*, B4094, and B4123. **(D)** Total lichenysin production (μg/ml) in skimmed milk for DSM13*^T^*, B4094, and B4123. The amount of lichenysin produced by all three strains in LB at selected time points as quantified with RP-HPLC-QTOF-ESI/MS. Gray circle or bar- DSM13*^T^*, white circle or bar- B4094, black circle or bar- B4123.

The growth of strains B4094, B4123, and DSM13*^T^* in LB and skimmed milk is presented in Figures 3A,B. The specific growth rate (μ, h^–1^), lag phase (λ), and the doubling time (Td) were calculated for each strain under different conditions [section “Estimation of Lag Time, Lambda (λ) and Specific Growth Rate, Mu (μ)”] and the results are shown in Table 2. Starting from an initial level of 1–2 log_10_ CFU/ml, each of the strains reached similar cell densities in a stationary phase of around 9 log_10_ CFU/ml within 12–14 h in LB (Figure 3A), with B4123 having the shortest lag time, and DSM13 having the highest growth rate (μ = 2.75 h^–1^; Td = ∼15 min) (Table 2).

**TABLE 2:** Estimated growth rate mu (μ) and lag time (λ) for DSM13*^T^*, B4094, and B4123 in LB and skimmed milk using the Baranyi primary growth model.

Medium	Strain	Parameter	logN_0_ (log_10_CFU/ml)	logN_max_ (log_10_CFU/ml)	μ (h^–1^)	λ (h)	Td (min)
LB	DSM13*^T^*	Estimate	1.8	8.9	2.75	5.88	15.1
		Standard Error	0.2	0.1	0.12	0.39	
	B4094	Estimate	1.4	8.8	2.05	4.51	20.3
		Standard Error	0.1	0.1	0.05	0.32	
	B4123	Estimate	0.7	8.9	1.93	1.70	21.5
		Standard Error	0.2	0.1	0.04	0.38	
Skimmed	DSM13*^T^*	Estimate	0.8	9.0	0.74	0.24	56.4
milk		Standard Error	0.4	0.2	0.02	1.66	
	B4094	Estimate	1.1	9.0	0.69	0.66	60.2
		Standard Error	0.3	0.2	0.01	1.32	
	B4123	Estimate	1.3	8.8	0.74	0.67	56.4
		Standard Error	0.2	0.1	0.01	1.00	

Lag times and growth rates in skimmed milk were similar for the three strains. All had shorter lag times and lower growth rates (μ∼ 0.7 h^–1^) than in LB and entered the stationary phase approximately 10 h later. Final cell densities of around 9 log_10_ CFU/mL were reached after 22–24 h incubation (Figure 3B). At this moment, the milk showed spoilage.

#### Lichenysin Production in LB Broth and Skimmed Milk

The total lichenysin concentrations during the growth of strains B4094, B4123, and DSM13*^T^* at 37 °C were determined in LB and skimmed milk. Lichenysin was not found in cultures of DSM13*^T^* in both media, with the exception of a very low level (0.1 μg/ml) in LB at 20 h (Figures 3C,D). For the two food isolates, lichenysin was not detected in LB and skimmed milk at cell densities < 5 log_10_ CFU/ml.

In LB, lichenysin was first detected at time point 10 h, namely, 0.2 μg/ml at a cell density of 7 log_10_ CFU/ml (strain B4094) and 5.4 μg/ml at a cell density of 8 log_10_ CFU/ml (strain B4123) (Figure 3C). Upon entering into stationary phase, lichenysin concentrations increased to 35.9 μg/ml and 19.1 μg/ml for strain B4094 and B4123, respectively, with cell densities of around 8–9 log_10_ CFU/ml (Figure 3C).

The production of lichenysin was significantly lower in skimmed milk than in LB for both strains, with B4094 producing more lichenysin than strain B4123 in both cases. In the milk, low lichenysin levels were first detected after 22 h for strain B4094, namely 0.2 μg/ml at a cell density of ∼7.9 log_10_ CFU/ml, with levels increasing to 8.1 μg/ml in the stationary stage (8.8 log_10_ CFU/ml) after 28 h of incubation (Figure 3D). For strain B4123, low levels of lichenysin (0.1 μg/ml) were first detected at 16 h, and levels reached only 0.8 μg/ml during the stationary phase at cell densities of ∼8.5 log_10_ CFU/ml (Figure 3D).

Lichenysin production and total viable counts were furthermore determined at two later time points (48 and 72 h), showing that cell concentrations in LB and skimmed milk did not change in cultures of B4094, B4123, and DSM13*^T^*, and the lichenysin concentrations did not increase (Supplementary Tables S4, S5).

It can be concluded from Figure 3 that (1) lichenysin was not detected in LB and skimmed milk at cell densities < 5 log_10_ CFU/ml; (2) lichenysin production started when cells reached the late exponential phase in LB and skimmed milk; (3) lichenysin production was strain and medium-dependent at similar cell densities.

#### Identification and Quantification of Lichenysin A Variants in LB Broth and Skimmed Milk

**FIGURE 4:**
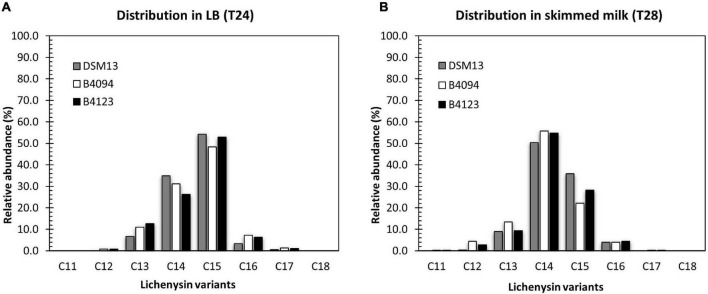
Distribution of lichenysin A variants, produced in LB broth and skimmed milk at 37°C. Relative abundance of individual lichenysin variants as part of the total amount in **(A)**- LB broth at 24 h; **(B)** skimmed milk at 28 h. Gray bar- DSM13*^T^*, white bar- B4094, black bar- B4123.

The concentrations of lichenysin as reported in the previous section consisted of the sum of lichenysin variants, all corresponding with lichenysin A variants. The molar masses of the detected variants are listed in Supplementary Table S1. The percentages of individual variants based on the total amount found are presented in Figures 4A,B for strains B4094, B4123, and DSM13*^T^* upon growth in LB for 24 h and in skimmed milk for 28 h. An example of chromatograms with mass spectra from the extracts of the B4094 cultured cells in skimmed milk as retrieved at 28 h is shown in Supplementary Figure S5.

The five main lichenysin A variants found were C12, C13, C14, C15, and C16 (molar mass of 993.7, 1007.7, 1021.7, 1035.7, and 1049.7 Da respectively). In addition, strains B4094, B4123 and DSM13*^T^* synthesized two other variants, namely, C11-lichenysin (979.6 Da) and C17-lichenysin (1063.7 Da). Lichenysin with the shortest hydrophobic tail (C11) was only detected in the case of the two food isolates (B4094 and B4123) grown in skimmed milk but not in LB. The relative abundance of lichenysin with the longest hydrophobic tail (C17) was higher in LB than in skimmed milk for all three strains (Figure 4 and Supplementary Table S4). The 14 Da mass shift between the molecular masses detected could arise from amino acid substitutions in the peptide moiety (e.g., valine vs. isoleucine) or different lengths of the fatty acid chain. Given the ∼1 min shorter elution time of the variant with the 14 Da lower mass than C12 (Supplementary Figure S5), this mass difference could be assigned to the acyl chain length. As a comparison, C15-surfactin and C15-lichenysin (differing in peptide ring but not chain length) had very similar retention times (∼13 min).

Approximately half of the main variants produced in LB consisted of C15-lichenysin, followed by C14 and smaller amounts of C13 > C16 > C12 (Figure 4A). In skimmed milk, on the other hand, C14-lichenysin had the highest relative abundance (around 55%), followed by C15 and smaller amounts of C13 > C16 > C12. This was observed for each of the three strains, indicating that the composition of the medium has a strong influence on the distribution of the chain length.

To further test whether the medium composition and culture conditions affect the levels of lichenysin produced and the synthesis of different isoforms, the total lichenysin production of B4094, B4123, and DSM13*^T^* on agar surfaces was also quantified.

### Surface Growth on LB Agar and Skimmed Milk Agar and Production of Lichenysin and Its Variants at Optimal Temperature (37°C) and High Temperature (55°C)

#### Lichenysin Production on LB Agar and Skimmed Milk Agar at 37 and 55°C

**FIGURE 5:**
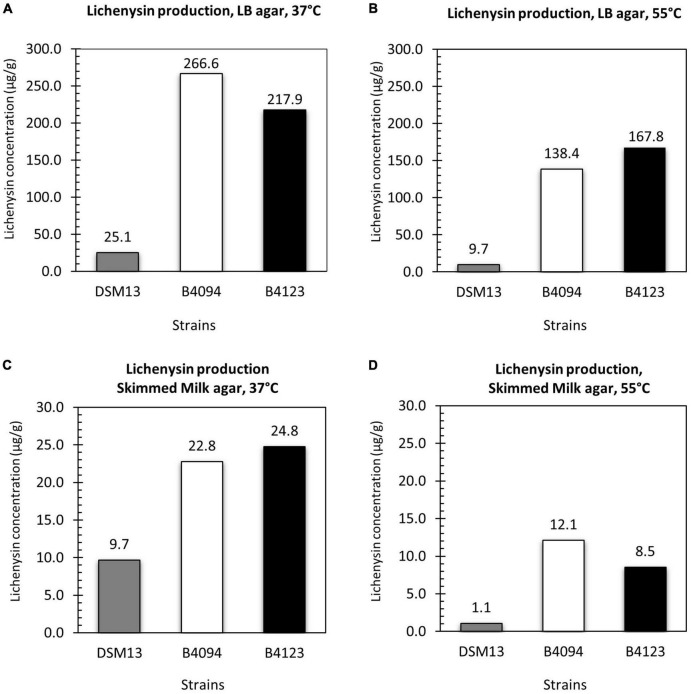
Lichenysin production upon surface growth on LB agar and skimmed milk at 37 and 55°C. Total lichenysin produced by DSM13*^T^*, B4094, and B4123 per g of wet biomass after 10 days of incubations on **(A)** LB agar at 37°C; **(B)** LB agar at 55°C; **(C)** skimmed milk agar at 37°C; **(D)** skimmed milk agar at 55°C. Gray bar- DSM13*^T^*, white bar- B4094, black bar- B4123.

The amounts and variants of lichenysin as produced by strains B4094, B4123, and DSM13*^T^* upon growth on LB agar and skimmed milk agar at 37 and 55°C, are presented in Figure 5. The amount of lichenysin produced by all three strains was measured at a late time point (after 10 days of incubation of the agar plates).

On LB agar at 37°C, DSM13*^T^*, B4094, and B4123 yielded 25 μg lichenysin per g of wet biomass (μg/g), 267 μg/g, and 218 μg/g, respectively (Figure 5A). All three strains also produced lichenysin at 55°C, but at lower concentrations than at 37°C, namely, ∼ 60 % less for DSM13*^T^*, ∼50 % less (138 μg/g) for B4094, and ∼30 % less (168 μg/g) for B4123 (Figure 5B).

Similar trends were observed upon growth on skimmed milk agar, albeit that the total concentrations produced on milk agar were around ten-fold lower than on LB agar. At 37°C, strains DSM13*^T^*, B4094, and B4123 produced 10 μg/g, 23 μg/g, and 25 μg/g of lichenysin per g of wet biomass, respectively (Figure 5C), and at 55°C the total lichenysin concentrations were between ∼50 and 90% lower (Figure 5D).

Similar to the results reported in section “Identification and Quantification of Lichenysin A Variants in LB Broth and Skimmed Milk,” B4094 and B4123 produced significantly higher amounts of lichenysin than the type strain DSM13 under all conditions tested.

#### Identification and Quantification of Lichenysin A Variants on LB Agar and Skimmed Milk Agar at 37 and 55°C

**FIGURE 6:**
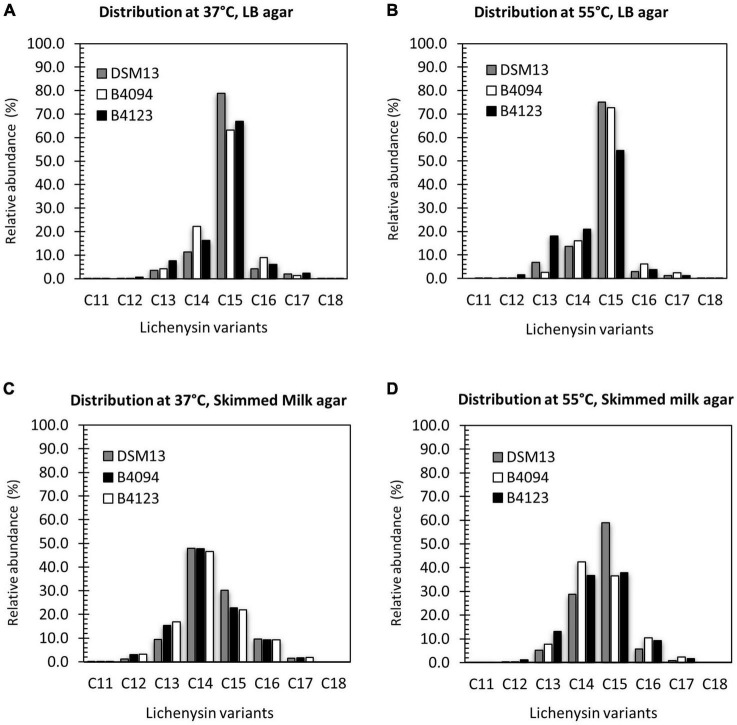
Distribution of lichenysin A variants upon surface growth on LB agar and skimmed milk agar at 37 and 55°C. Relative abundance of individual lichenysin variants as part of the total amount extracted from biomass of DSM13*^T^*, B4094, and B4123 after 10 days of incubations on **(A)** LB agar at 37°C; **(B)** LB agar at 55°C; **(C)** skimmed milk agar at 37°C; **(D)** skimmed milk agar at 55°C. Gray bar- DSM13*^T^*, white bar- B4094, black bar- B4123.

The distribution of lichenysin A variants upon production on LB agar and skimmed milk agar at 37 and 55°C is presented in Figure 6. Upon growth on LB agar at 37 and 55°C, all three strains produced C15 as the dominant variant (in most cases > 60%), followed by the variants C14 > C13 > C16 > C12-lichenysin. The relative abundance of the C17 variant was low but similar for the three strains (Figures 6A,B).

On skimmed milk agar, C14- lichenysin had the highest relative abundance at 37°C for all three strains, followed by C15 > C13 > C16-lichenysin (Figure 6C). At 55°C, the C14 and C15 variants made up 70–90% of the total lichenysin, with similar ratios for strains B4094 and B4123, but two times higher levels of C15 than C14 for DSM13*^T^*. At this high temperature, C13 and C16- lichenysin each constituted around 10% of the total amount, with only very low levels of C17 and the C12-variant (Figure 6D).

A variant with the longest hydrophobic tail (C18) was found to be produced at very low levels by all three strains at 37°C on LB agar but not on skimmed milk agar (Figure 6 and Supplementary Table S6). The C18 variant (with ∼1 min higher elution time) was detected in biomass of B4094 obtained after growth at 55°C on LB agar, but not for the other two strains growth at 55°C on LB. In contrast, the shortest C11 variant was detected only in skimmed milk or skimmed milk agar, but not in LB broth or on LB agar at 37°C and 55°C (Supplementary Table S6). However, one exception was observed, as the B4123 strain produced the C11 variant on LB agar at 55°C. Together, these results indicate that the tail lengths of the lichenysin variants and their relative abundances depend on the strain, cultivation medium, and temperature.

Considering the above outcomes that culturing conditions can strongly influence the amount and type of lichenysin produced, lichenysin production was also evaluated upon culturing of DSM13*^T^*, B4094, and B4123 in liquid broth using stagnant conditions. Results presented in Supplementary Figure S6 show that lichenysin concentrations were significantly higher in the fractions that contained cells (pellicle and cell pellet) compared with the supernatant, with the highest amount observed in the pellicle that was formed at the liquid-air interface.

### Lichenysin Is Slightly More Toxic to Caco-2 Cells Than Surfactin

**FIGURE 7:**
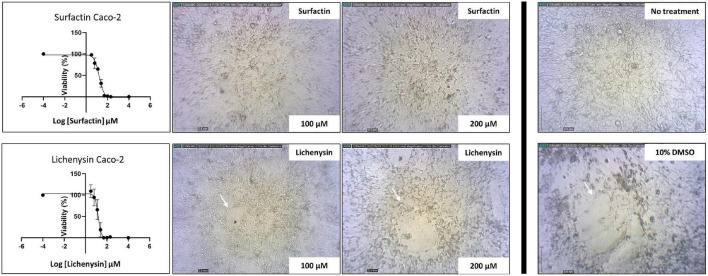
Toxicity of lichenysin and surfactin toward human Caco-2 cells. Caco-2 cells were seeded (2 × 10^5 cells/ml) in 96 wells-plates and exposed to different lichenysin and surfactin concentrations (only 100 and 200 μM are shown; in log_10_ scale = 2 and 2.3 μM, respectively). No-treatment cells were the negative control and 10% DMSO was used as a control for cell death. Cell viability was monitored for 72 h, microscopic pictures of cell disruption for lichenysin after 24 and 72 h are shown. The IC_50_ for lichenysin is 16.6 μM (=∼16.6 μg/ml), in log_10_ scale = 1.22 μM; and for surfactin is 23.5 μM (=∼25.5 μg/ml), in log_10_ scale = 1.37 μM.

The toxicity of lichenysin toward human cells and pig organoids was investigated, using *B. subtilis* derived surfactin as a control. The concentration of lichenysin and surfactin needed to reduce cell viability by 50% (IC_50_) is presented for different cell lines and organoids in Table 3. Lichenysin and surfactin were not toxic to HEK293 embryonic kidney cells at the highest tested concentration of 200 μM (∼200 μg/ml), even after prolonged exposure for 72 h. Lichenysin and surfactin led to a reduction of the viability of Caco-2 cells after 72 h incubation with IC_50_ values of 16.6 μg/ml and 23.4 μg/ml, respectively (Figure 7). Cells exposed to lichenysin or surfactin at 100 μM (∼100 μg/ml) or 200 μM (∼200 μg/ml) showed different degrees of cell impairment indicated by reduced fluorescence in the Alamar Blue assay and cells incubated with the solvent DMSO alone at 10% w/v showed severe impairment (Figure 7). The viability of the pig organoids upon exposure to lichenysin and surfactin was also determined, showing IC_50_ for lichenysin of 16.8 μg/ml, while surfactin at the highest tested concentration of 200 μM did not show toxicity, indicating an IC_50_ > 200 μg/ml.

**TABLE 3:** Cytotoxic level of lichenysin and surfactin (IC_50_) to human and mammalian cells.

Cell line	Surfactin IC_50_ (μg/g)	Lichenysin IC_50_ (μg/g)
HEK293	>200	>200
Caco-2	23.5	16.6
Pig ileum organoids	>200	16.8

## Discussion

The results presented in this study showed that nine out of ten *B. licheniformis* food isolates have robust growth properties. The amount of lichenysin being produced by the isolates was dependent on the strain, cultivation medium, and temperature of incubation. Lichenysin showed toxic effects in pig ileum organoids and human epithelial Caco-2 cells.

Strain B4094 consistently produced more lichenysin than strain B4123 under all conditions tested, and both high producers synthesized much more lichenysin than DSM13*^T^*. The observed variation between strains is in line with an earlier report by Madslien et al. (2013), who demonstrated that the amounts produced by 53 *B. licheniformis* strains varied more than two orders of magnitude (ranging from <0.013 μg to >3.3 μg per mg biomass) between strains. Some high producers in their study were associated with foodborne intoxications and they also classified strain ATCC14580 (=DSM13*^T^*) as a weak producer.

The analysis of lichenysin production in liquid LB or skimmed milk (as a food matrix) revealed that lichenysin was not detected at cell densities < 5 log_10_ CFU/ml. Production did not reach maximum levels until the stationary stage in both media. It is known that lichenysin production can be influenced by environmental factors, e.g., medium composition (Yakimov et al., 1996), oxygen availability, temperature, and pH (Yakimov et al., 1995; Joshi et al., 2008; Li et al., 2008; Gogotov and Miroshnikov, 2009), and the type of carbon source (important for energy generation) or nitrogen sources (for amino acids and protein synthesis). Reported yields of lichenysin were for instance higher in cultivation media containing glucose than in media with other carbon sources like sucrose, maltose, glycerol, sodium citrate, sodium acetate, lactose, corn starch, and starch (Yakimov et al., 1995; Qiu et al., 2014). In addition, the yield increased in the presence of specific amino acids like L-glutamic acid and L-asparagine in the medium (Yakimov et al., 1996) and when inorganic nitrogen sources [such as KNO_3_, NaNO_3_, NH_4_Cl, (NH_4_)_2_SO_4_, and NH_4_NO_3_] were present instead of organic nitrogen sources (peptone, yeast extract, etc.) were present (Qiu et al., 2014). LB contains glucose and readily available nitrogen sources (yeast extract and peptone), whereas skimmed milk contains the sugar lactose and casein as a nitrogen source, requiring proteolytic cleavage prior to uptake by cells. These differences in composition likely account for the significantly lower levels of lichenysin produced in milk than in LB as found in this study.

We furthermore showed that lichenysin yields were temperature-dependent, with lower yields at 55°C than at 37°C in biomass obtained from agar plates (both LB and milk agar) (Figure 5). This result is consistent with the study of Yakimov et al. (1995), who showed that lichenysin production by *B. licheniformis* is optimal at 35–45°C. Similarly, surfactin yield from *B. subtilis* was also influenced by temperature, with lower production at 45°C than at 35°C in Park et al. (2019) and higher yields at 37°C than at 25°C (Abdel-Mawgoud et al., 2008; Rahman and Ano, 2009). In another study, the production of surfactin by *Bacillus amyloliquefaciens* was however lower at 25°C or 30°C than at 15°C (Monteiro et al., 2016).

In our study, cultures contained lichenysin A variants with different acyl chain lengths (C11–C18). The length of the fatty acid chain lengths may influence the toxicity of the compound; Previous studies on surfactin showed that C15 acyl chains had more efficient penetration strength into the cell membrane than its shorter counterparts C13 and C14-surfactin (Liu et al., 2010; Wu et al., 2017), and exhibited enhanced antifungal and antibacterial activity (Meena and Kanwar, 2015). This is thought to result from more efficient interactions of a longer fatty acid chain of surfactin with the acyl chains of phospholipids in the cell membrane. Cultures containing lichenysin with a skew toward long-chain variants (which is medium and culture condition dependent) may therefore be more toxic to different cells than cultures with a higher proportion of short-chain variants.

The concentration of lichenysin needed to reduce cell viability by 50% (IC_50_) was 16.6 μg/ml for Caco- 2 human intestinal epithelial cells and 16.8 μg/ml for pig ileum organoids. These levels correspond with concentrations resulting in toxic effects on boar spermatozoa cells (i.e., >10 μg/ml) (Madslien et al., 2013). For surfactin, the IC_50_ value was 23.5 μg/ml for Caco-2 cells while no toxicity was seen for the ileum organoids at the highest levels tested (>200 μg/ml). This indicates that lichenysin is more toxic to these cell types than surfactin. In the case of surfactin, the purchased compound largely contained C15 acyl chains (>99%). The lichenysin extracts used in the toxicity assays contained a mix of lichenysin variants, with C15-lichenysin being the most abundant (>65%, produced by B4094 on LB agar 37°C, see Figure 6). In addition to differences in the peptide ring between surfactin and lichenysin, the presence of longer acyl chains (∼10% C16) may have contributed to the observed differences in toxicity toward the organoids. A further detailed mechanistic study with pure lichenysin variants having different carbon chain lengths would be required to elucidate this, however, such compounds are not commercially available.

Overall, levels of *B. licheniformis* up to 5 log_10_ viable cells/ml in liquid foods are unlikely to pose a foodborne hazard related to the presence of lichenysin. However, at higher cell densities, a risk of foodborne intoxication may arise due to the production of the compound. Lichenysin levels produced in milk and LB were in the same range as the IC_50_ values of lichenysin for epithelial cells. The actual amounts to cause illness *in vivo* upon ingestion of foods will depend on various factors, most notably the amount of food ingested and the concentration of the lichenysin in the product. High producers are commonly found in foods: in the study by Madslien et al. (2013), 28 out of 53 isolates produced > 3.3 μg/mg biomass on tryptic soy agar after 10 days at 37°C. The highest level found in our study was 0.27 μg/mg biomass for B4094 on LB agar. This 10-fold difference indicates that production of higher levels than reported in this study is conceivable in food products with high levels of *B. licheniformis*, depending on the strain present, the composition of the food and conditions during processing and storage in the chain. In solid foods, the distribution of the organism in the product may be inhomogeneous with ‘hot spots’ of high cell concentrations and lichenysin production. In confirmed foodborne intoxication cases due to consumption of solid product contaminated with *B. licheniformis*, high levels of the organism were observed (i.e., 2 × 10^6^ CFU/g in curried chicken and mayonnaise sandwich; 1 × 10^8^ CFU/g in minced beef pie; 1.1 × 10^8^ CFU/g in pancake) (Salkinoja-Salonen et al., 1999).

In addition to natural contamination of foods, *B. licheniformis* can be actively introduced into the food chain (e.g., as crop bioprotectants or feed/food additives). This is usually done in the form of spores. From a food processing point of view, it is important that the selected strains do not possess genetic elements that enable the production of extremely heat-resistant spores (Berendsen et al., 2016a,b), resulting in potential non-sterility issues in finished products.

From a food safety point of view, the production of lichenysin must be prevented, requiring a thorough assessment of surfactant production capacity. Once lichenysin is produced in food upon the growth of *B. licheniformis*, the compound is stable even when cells (or even spores) are inactivated. This stresses the importance of the control of this organism throughout the entire production chain, by inactivation of viable cells and spores in ingredients and prevention of outgrowth using appropriate preservation systems and/or low-temperature control.

## Data Availability Statement

The original contributions presented in the study are included in the article/Supplementary Material, further inquiries can be directed to the corresponding author/s.

## Ethics Statement

The animal study was reviewed and approved by Animal Ethics Committee of Wageningen University and Research. Written informed consent was obtained from the owners for the participation of their animals in this study.

## Author Contributions

KY designed and conducted the majority of the experiments, analyzed the results, and wrote the manuscript. MP performed the screening of *B. licheniformis* strains, hemolysis test, and anaerobic growth experiments. BF-C performed the alamarBlue cytotoxicity assay and took microscopic pictures. KY, TA, and MW-B discussed the experimental design and results. JW, TA, and MW-B reviewed the manuscript. All authors contributed to the article and approved the submitted version.

## Conflict of Interest

The authors declare that the research was conducted in the absence of any commercial or financial relationships that could be construed as a potential conflict of interest.

## Publisher’s Note

All claims expressed in this article are solely those of the authors and do not necessarily represent those of their affiliated organizations, or those of the publisher, the editors and the reviewers. Any product that may be evaluated in this article, or claim that may be made by its manufacturer, is not guaranteed or endorsed by the publisher.

## Footnotes

1
https://CRAN.R-project.org/package=biogrowth

## Abbreviations

ON: overnight
CMC: critical micelle concentration
DMEM: Dulbecco’s Modified Eagle Medium
CFU: colony-forming unit.
