# Supplementary material for: Lichenysin Production by Bacillus licheniformis Food Isolates and Toxicity to Human Cells
Source: Front Microbiol. 2022 Feb 7;13:831033. doi: 10.3389/fmicb.2022.831033 (PMC8859269; doi:10.3389/fmicb.2022.831033)

Supplementary Figure S1- Growth of different *Bacillus licheniformis* strains in LB medium under different conditions

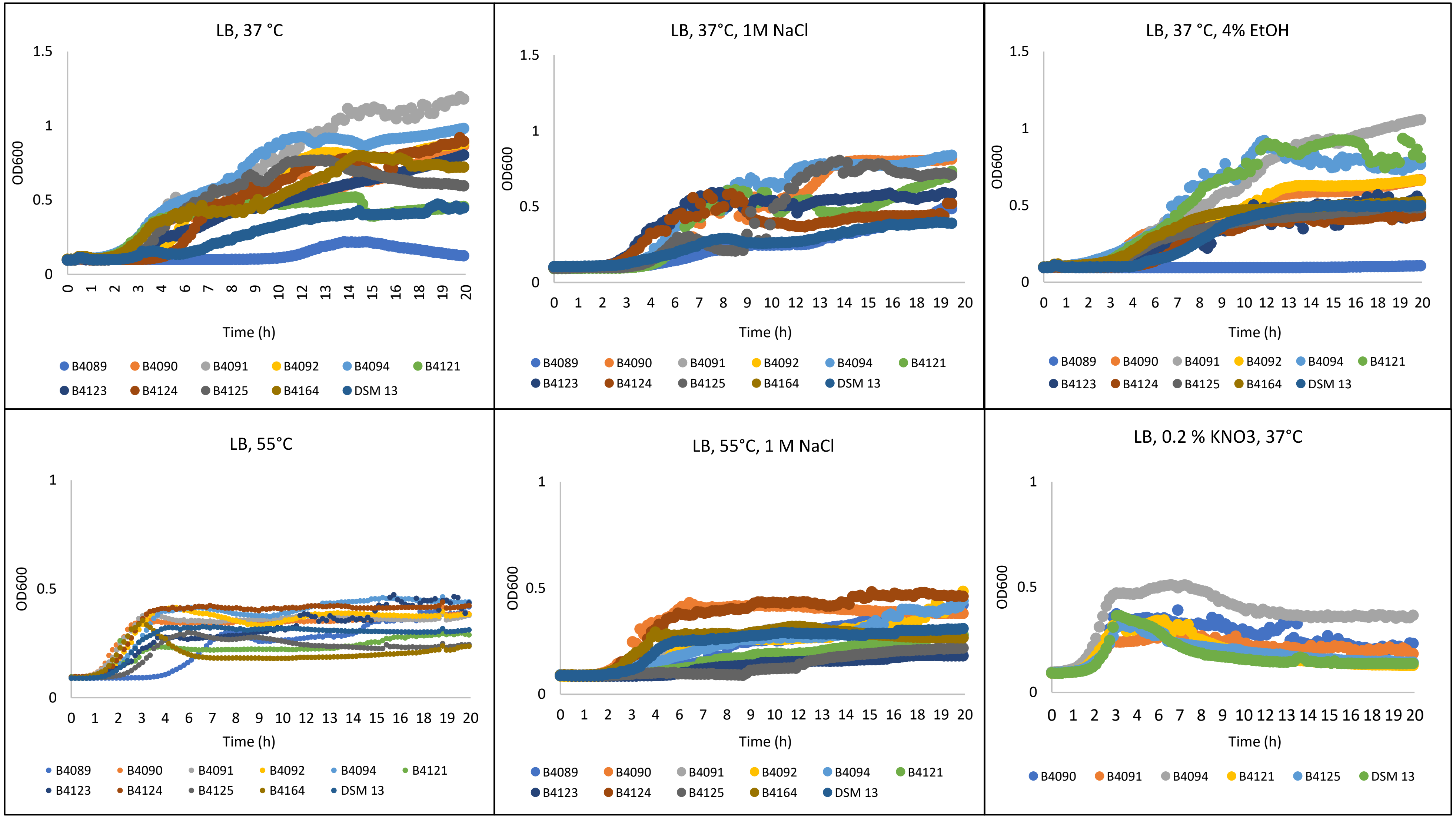

Supplementary Figure S2- Growth of different *Bacillus licheniformis* strains on agar plate, at 37°C, 55°C and 60°C

The OD_{600nm} of the ON cultures in LB media was measured and used to inoculate 5 ml of fresh LB medium to the OD_{600nm} 0.05. Cultures were then grown at 37 °C, 200 rpm until OD_{600nm} 0.5. This step was performed to get endospores-free culture. 1 ml of cells from the day culture in LB medium were collected by centrifugation at 10000 x g for 2 min (5430 R Eppendorf centrifuge) and resuspended in 1 ml sterile 0.9 % NaCl solution. The OD_{600nm} of the cells was measured and was adjusted to 1.0 with the 0.9 % NaCl solution. A 10-fold dilution series in the NaCl solution up to 10^{-9} was prepared. 3 µl of cells from each dilution tube was spotted on LB agar plates and dried under the flow cabinet. The plates were incubated at 37 °C, 55 °C, and 60 °C.

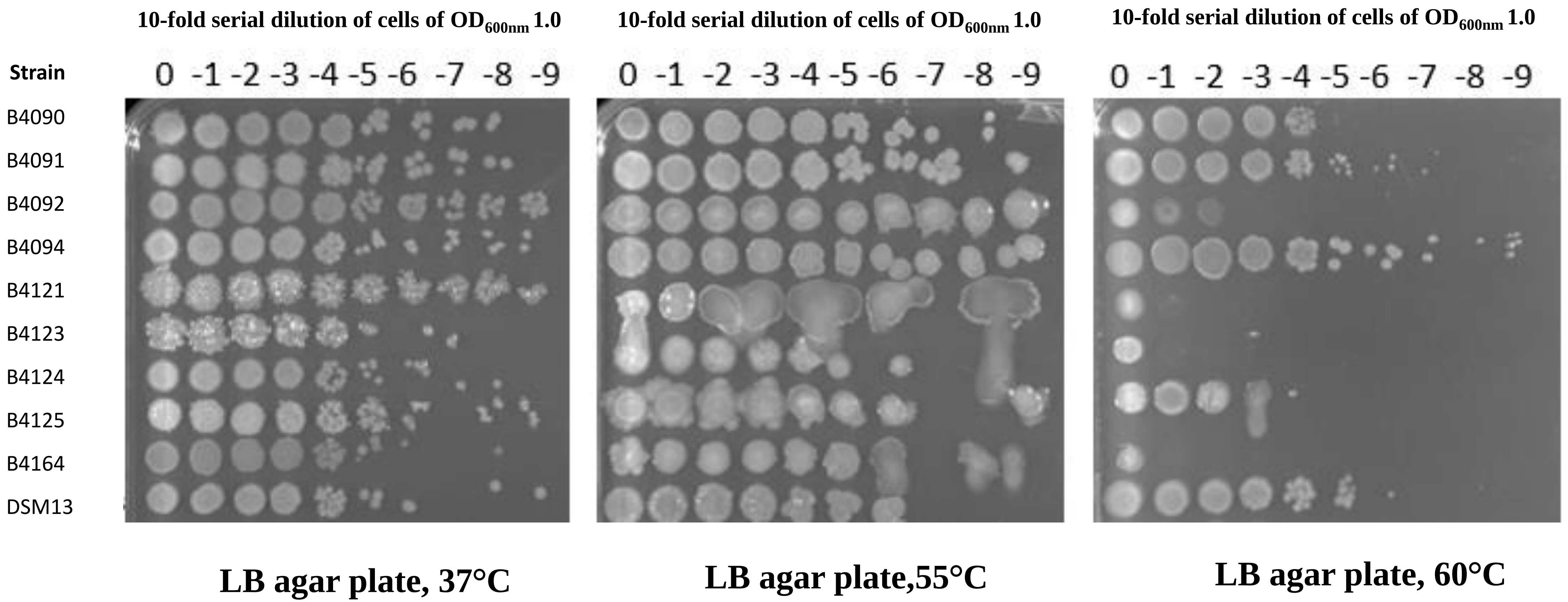

Supplementary Figure S3A- Hemeolysis Test for different *Bacillus licheniformis* strains

Growth of all 10 *B. licheniformis* food isolates and the type strain DSM13^{T} on Columbia blood agar, showing hemolytic activity. The clearing zone on the blood agar indicates the lysis of erythrocytes, indicative of the presence of the biosurfactant lichenysin. A small clearing zone indicated weak hemolytic activity and a big clearing zone indicated strong hemolytic activity.

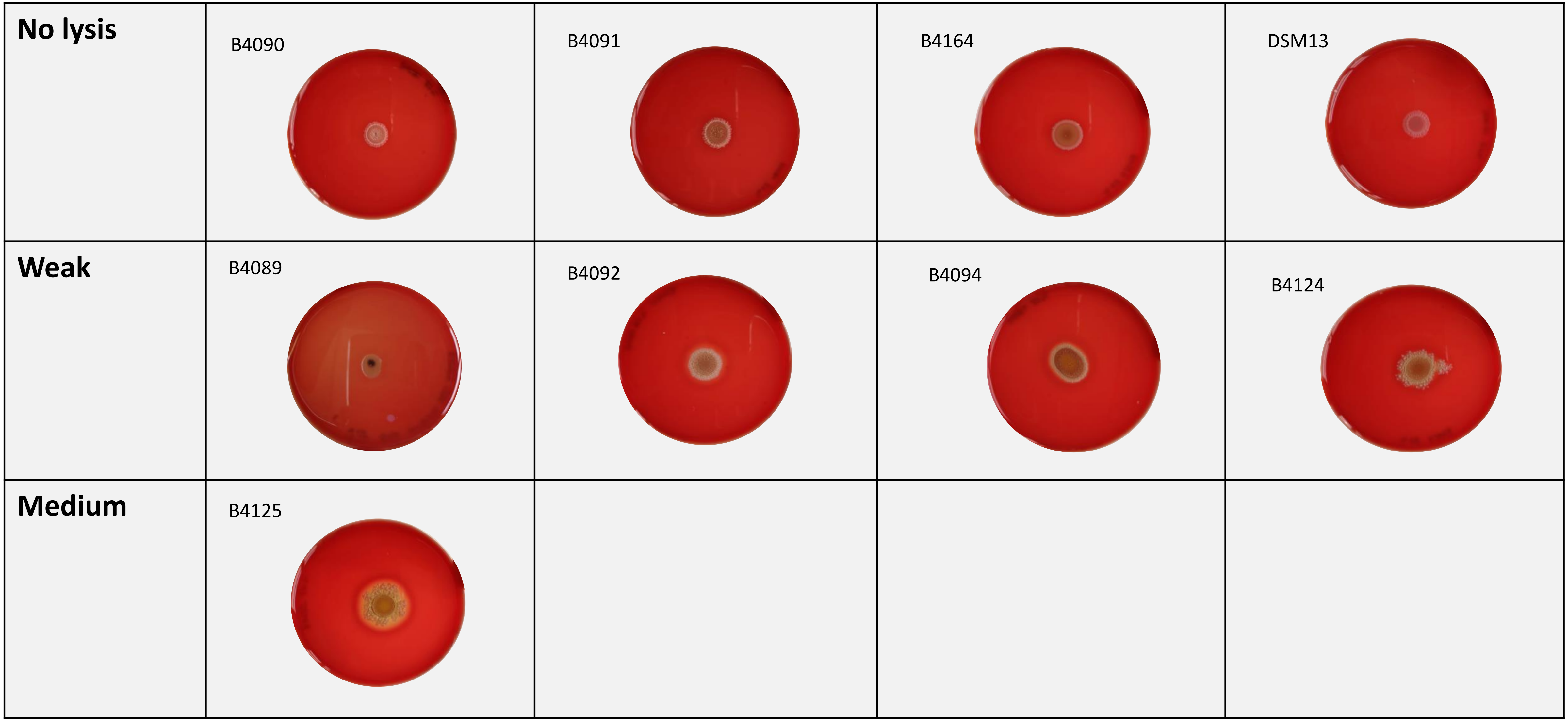

Supplementary Figure S3B- Hemeolysis Test for different *Bacillus licheniformis* strains

Growth of all 10 *B. licheniformis* food isolates and the type strain DSM13^{T} on Columbia blood agar, showing hemolytic activity. The clearing zone on the blood agar indicates the lysis of erythrocytes, indicative of the presence of the biosurfactant lichenysin. A small clearing zone indicated weak hemolytic activity and a big clearing zone indicated strong hemolytic activity.

**Strong**	B4121 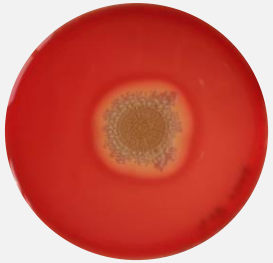			
**Super strong**	B4123 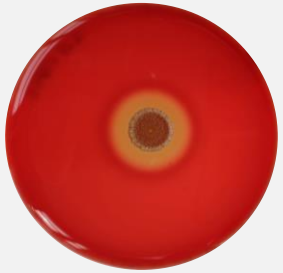			

Supplementary Figure S4 – Growth of different *Bacillus licheniformis* strains in TSB medium under different conditions

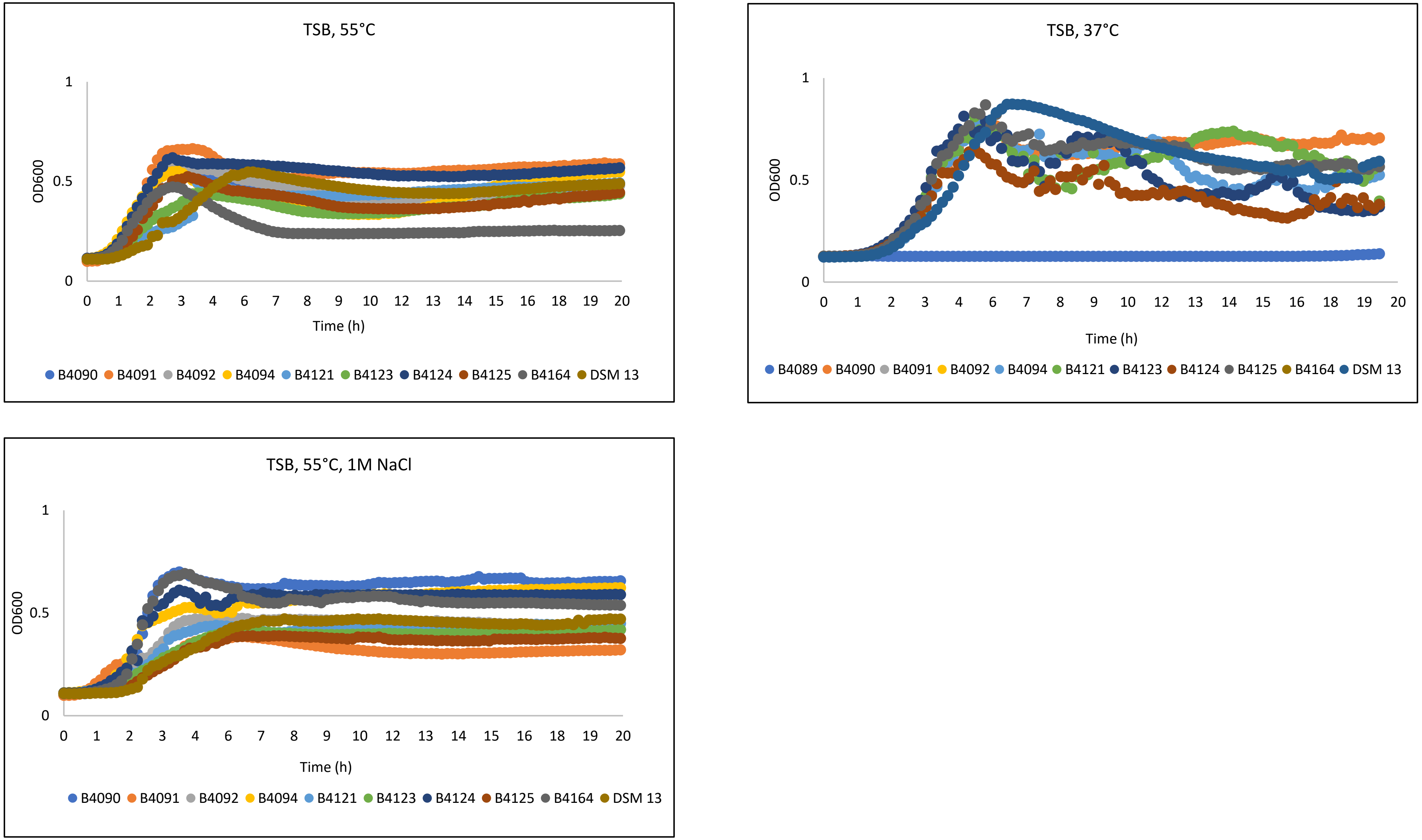

Figure S5- Chromatographs of lichenysin A variants

Chromatographs of C11 to C17- lichenysin A variants detected for B4094 via RP-HPLC-QTOF-ESI/MS. 5 µL sample was injected on a C8 analytical column (Phenomenex) thermostated at 50° C. Lichenysin A was eluted at a flow rate of 0.2 mL/min with a linear gradient of 0.10% formic acid in 40% water + 55% acetonitrile + 5% tetrahydrofuran to 0.1% formic acid in 75 % acetonitrile + 25% tetrahydrofuran in 20 min. Lichenysin A variants were screened according to the masses in Table S1. Quantitative analyses were carried out using lichenysin A standard (range 10 – 40 000 µg/L) (Lipofabrik).

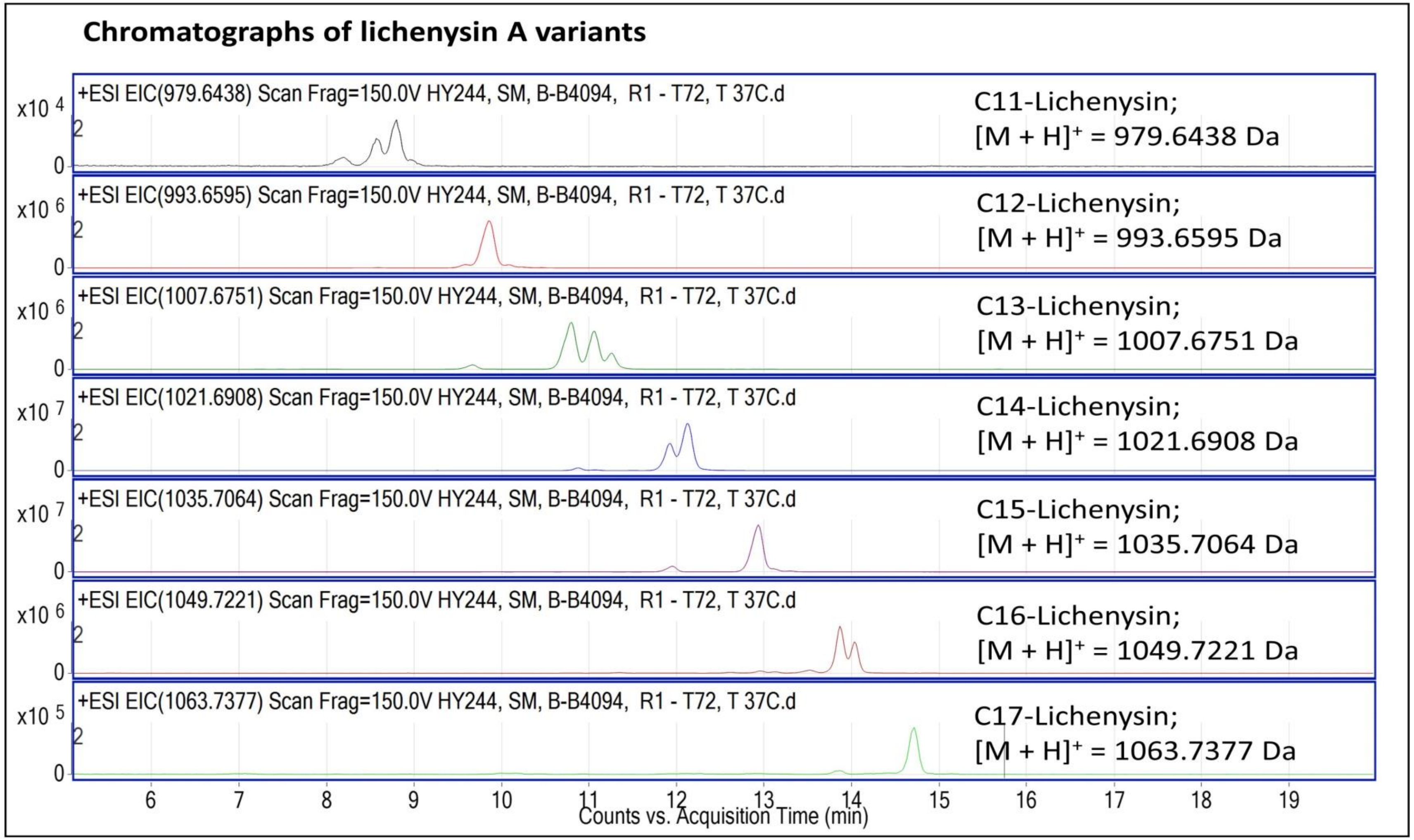

Figure S6- *Bacillus licheniformis* growth and lichenysin production in different matrices

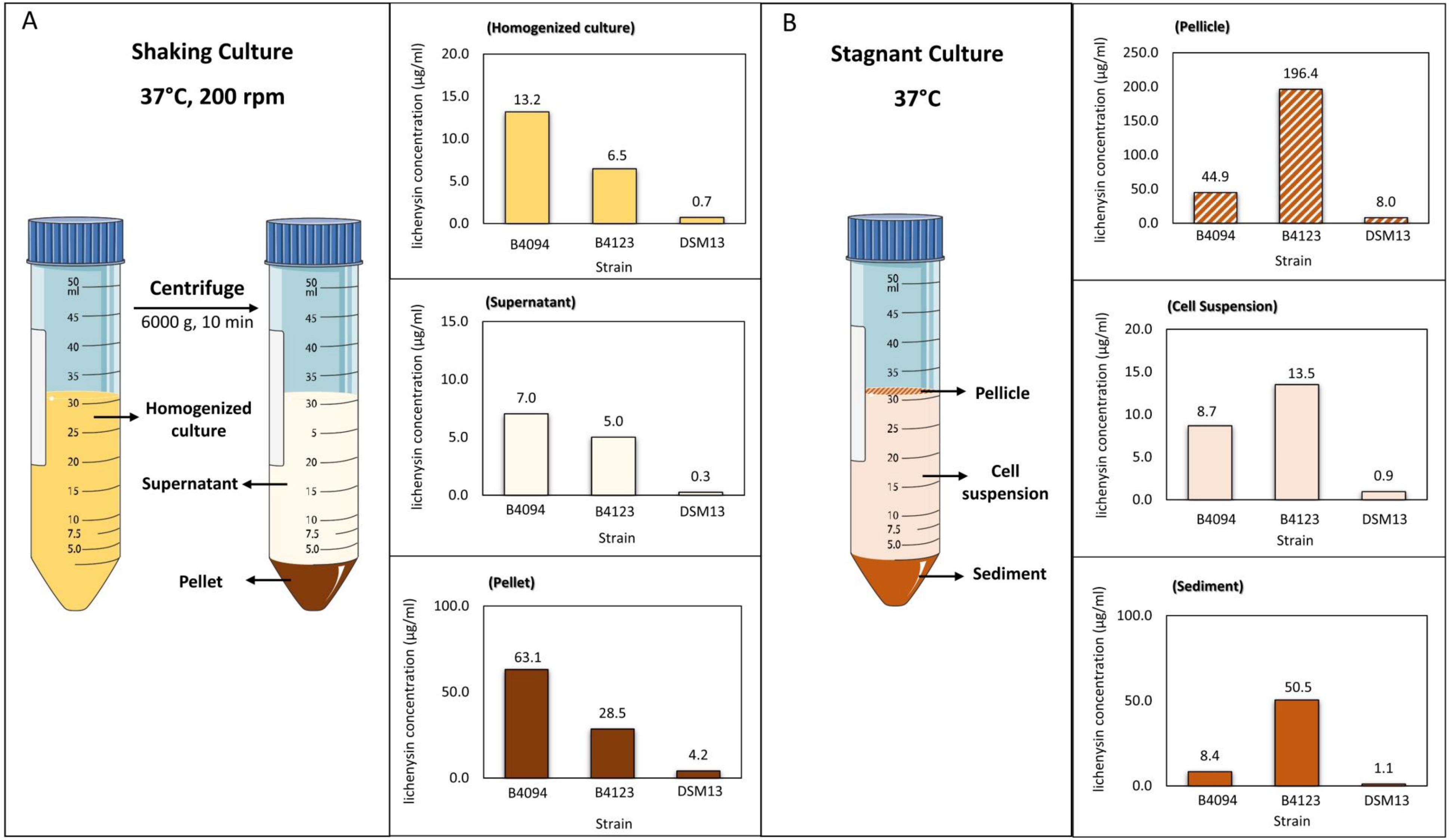

Supplement: Supplementary file 1 [file Data_Sheet_1.PDF]
