# Supplementary material for: Lichenysin Production by Bacillus licheniformis Food Isolates and Toxicity to Human Cells
Source: Front Microbiol. 2022 Feb 7;13:831033. doi: 10.3389/fmicb.2022.831033 (PMC8859269; doi:10.3389/fmicb.2022.831033)
Supplement: Supplementary file 3 [file Table_2.DOCX]

**Supplementary Figures**

**Supplementary Figure S1: Growth of different *Bacillus licheniformis* strains in LB medium under different conditions**

**Supplementary Figure S2: Growth of different *Bacillus licheniformis* strains on agar plates, at 37°C, 55°C, and 60°C.** The OD_600nm_ of the ON cultures in LB media was measured and used to inoculate 5 ml of fresh LB medium to the OD_600nm_ 0.05. Cultures were then grown at 37 °C, 200 rpm until OD_600nm_ 0.5. This step was performed to get endospores-free culture. 1 ml of cells from the day culture in LB medium were collected by centrifugation at 10000 x g for 2 min (5430 R Eppendorf centrifuge) and resuspended in 1 ml sterile 0.9 % NaCl solution. The OD_600nm_ of the cells was measured and was adjusted to 1.0 with the 0.9 % NaCl solution. A 10-fold dilution series in the NaCl solution up to 10^-9^ was prepared. 3 µl of cells from each dilution tube was spotted on LB agar plates and dried under the flow cabinet. The plates were incubated at 37 °C, 55 °C, and 60 °C.

**Supplementary Figure S3: Hemolysis test for different *Bacillus licheniformis* strains**

Growth of all 10 *B. licheniformis* food isolates and the type strain DSM13^T^ on Columbia blood agar, showing hemolytic activity. The clearing zone on the blood agar indicates the lysis of erythrocytes, indicative of the presence of the biosurfactant lichenysin. A small clearing zone indicated weak hemolytic activity and a big clearing zone indicated strong hemolytic activity.

**Supplementary Figure S4: Growth of different *Bacillus licheniformis* strains in TSB medium under different conditions**

**Supplementary Figure S5: Chromatograph of lichenysin A variants**

Chromatographs of C11 to C17- lichenysin A variants detected for B4094 via RP-HPLC-QTOF-ESI/MS. 5 µL sample was injected on a C8 analytical column (Phenomenex) thermostated at 50°C. Lichenysin A was eluted at a flow rate of 0.2 mL/min with a linear gradient of 0.10% formic acid in 40% water + 55% acetonitrile + 5% tetrahydrofuran to 0.1% formic acid in 75 % acetonitrile + 25% tetrahydrofuran in 20 min. Lichenysin A variants were screened according to the masses in Table S1. Quantitative analyses were carried out using lichenysin A standard (range 10 – 40 000 µg/L) (Lipofabrik).

**Supplementary Figure S6- *Bacillus licheniformis* growth and lichenysin production in different matrix**

**Supplementary Tables**

Supplementary Table S1: Molar masses of lichenysin A variants

Supplementary Table S2: Lichenysin production screening in biomass obtained from LB agar

Supplementary Table S3: The presence/absence of the lichenysin gene cluster

Supplementary Table S4: Lichenysin production and variant distribution in LB broth and skimmed milk

Supplementary Table S5: Cell concentration (log10 CFU/ ml) and total lichenysin production (µg/ml) at T48 and T72 h in LB medium and skimmed milk

Supplementary Table S6: Lichenysin production and variant distribution in biomass obtained from LB agar and skimmed milk agar
